# Plasma Aβ42 and Total Tau Predict Cognitive Decline in Amnestic Mild Cognitive Impairment

**DOI:** 10.1038/s41598-019-50315-9

**Published:** 2019-09-27

**Authors:** Ting-Bin Chen, Yi-Jung Lee, Szu-Ying Lin, Jun-Peng Chen, Chaur-Jong Hu, Pei-Ning Wang, Irene H. Cheng

**Affiliations:** 1 Institute of Brain Science, National Yang-Ming University, Taipei, Taiwan; 2 Department of Neurology, Neurological Institute, Taichung Veterans General Hospital, Taichung, Taiwan; 3 Dementia and Parkinson’s Disease Integrated Center, Taichung Veterans General Hospital, Taichung, Taiwan; 4 Center for Geriatrics and Gerontology, Taichung Veterans General Hospital, Taichung, Taiwan; 5 Division of Neurology, Department of Medicine, Taipei City Hospital Renai Branch, Taipei, Taiwan; 6 Taipei Municipal Gan-Dau Hospital, Taipei, Taiwan; 7 Biostatistics Task Force of Taichung Veterans General Hospital, Taichung, Taiwan; 8 Department of Neurology, Shuang Ho Hospital, Taipei Medical University, New Taipei City, Taiwan; 9 Division of General Neurology, Department of Neurological Institute, , Taipei Veterans General Hospital, Taipei, Taiwan; 10 Aging and Health Research Center, National Yang-Ming University, Taipei, Taiwan; 11 Brain Research Center, National Yang-Ming University, Taipei, Taiwan

**Keywords:** Predictive markers, Alzheimer's disease

## Abstract

Levels of amyloid-β (Aβ) and tau peptides in brain have been associated with Alzheimer disease (AD). The current study investigated the abilities of plasma Aβ42 and total-tau (t-tau) levels in predicting cognitive decline in subjects with amnestic mild cognitive impairment (MCI). Plasma Aβ42 and t-tau levels were quantified in 22 participants with amnestic MCI through immunomagnetic reduction (IMR) assay at baseline. The cognitive performance of participants was measured through neuropsychological tests at baseline and annual follow-up (average follow-up period of 1.5 years). The predictive value of plasma Aβ42 and t-tau for cognitive status was evaluated. We found that higher levels of Aβ42 and t-tau are associated with lower episodic verbal memory performance at baseline and cognitive decline over the course of follow-up. While Aβ42 or t-tau alone had moderate-to-high discriminatory value in the identification of future cognitive decline, the product of Aβ42 and t-tau offered greater differential value. These preliminary results might suggest that high levels of plasma Aβ42 and t-tau in amnestic MCI are associated with later cognitive decline. A further replication with a larger sample over a longer time period to validate and determine their long-term predictive value is warranted.

## Introduction

Mild cognitive impairment (MCI) refers to a transitional state on the continuum of cognitive function between normal aging and mild dementia. Amnestic MCI, characterized by an isolated deficit in episodic memory accompanied by intact general cognitive functioning, has been associated with biomarkers for Alzheimer disease (AD) and is now recognized as a risk factor for AD^1–4^. Early identification of subsequent cognitive decline in MCI patients is critical for prompt clinical intervention and therapeutic options. Thus, seeking validated biomarkers for risk of cognitive decline is crucial.

To date, validated biomarkers associated with AD include measurement of brain Aβ deposition presented by Aβ42 levels in cerebrospinal fluid (CSF), cortical amyloidosis on positron emission tomography (PET), measures of AD-typical neurodegeneration indicated by total-tau (t-tau) and phosphorylated-tau levels in CSF, hypometabolism on PET, and cerebral atrophy on magnetic resonance imaging (MRI)^5^. Increasing evidence supports an ordered, sequential, non-paralleled temporal trajectory of these five central biomarkers over many years in the continuum of AD^6,7^. In addition, these biomarkers play significant roles in the definition of AD pathology^5,8^. However, many obstacles, including invasiveness, time, cost, and accessibility to health care services, limit the widespread use of these biomarkers in the clinical setting. Rapid, minimally-invasive, and low-cost blood-based biomarkers are needed to assist in the identification of underlying pathophysiological processes involved in the early stages of AD and in longitudinal tracking of various disease indicators of progression of AD pathology.

Aβ accumulation and neurofibrillary tangle formation of phosphorylated-tau precedes full-blown clinical symptoms of AD by many years to decades, while Aβ deposition plateaus when patients progress into the clinical MCI phase of AD^9^. At this stage of the disease, neurofibrillary tangle formation, increasing gliosis, and progressive neuronal loss are initiated and continue to progress into the clinical state of overt dementia^9^. Accumulating evidence supports that various clearance mechanisms of toxic aggregation of misfolded peptides from the brain to the blood account for a determinate amount of Aβ and tau in the blood, and therefore, the measurability of low levels of these peptides is only attainable with highly sensitive, robust, and accurate assays^10–12^. Recent researches using ultrasensitive bioanalytic techniques have demonstrated that peripheral and central markers of amyloidosis and neurodegeneration are closely correlated^12–15^. Newly-developing analytic technologies may provide increasing clinical access to simple, convenient, and accurate blood-based biomarkers for high-risk individuals.

Immunomagnetic reduction (IMR) assay is a relatively new high-sensitivity detection technology for the quantification of Aβ and tau. IMR involves antibody-functionalized magnetic nanoparticles in biofluids to measure the reduction in magnetic signals through use of an ultra-high-sensitivity magneto-susceptometer, a superconducting quantum interference device (SQUID)^16^. Previous SQUID-based IMR studies have found that the utilization of a combination of plasma Aβ42 and total tau (t-tau) levels can reliably and accurately identify AD patients in both the prodromal and dementia stage^16–18^. However, the plasma concentrations of Aβ42 and t-tau in patients with amnestic MCI have not yet been thoroughly characterized using IMR assays.

The current study used IMR to investigate the predictive value of plasma levels of Aβ42 and t-tau in cognitive decline in subjects with amnestic MCI.

## Methods

### Subjects

The current prospective case-control study was conducted from 2015 to 2017. Study participants were consecutively recruited from the memory clinics at Taipei Veterans General Hospital and Shuang Ho Hospital in Taiwan. The inclusion criteria were age of ≥60 years and consent to a longitudinal follow-up period of clinical, neuropsychological, and brain MRI examinations. Prior to testing, written informed consent was obtained from all participants (or their legal guardians) for publication of this research article. The local institutional review board and the ethics committee of Taipei Veterans General Hospital and Shuang Ho Hospital approved the data collection protocol. We confirm that all methods were performed in accordance with the relevant guidelines and regulations by including a statement in the methods section to this effect. All authors have approved the manuscript for submission and gave consent for publication.

### Neuropsychological evaluations

All participants were subjected to a standard battery of clinical and comprehensive neuropsychological assessments at baseline and during annual follow-ups. The Mini-Mental Screening Examination (MMSE)^19^ and the Clinical Dementia Rating (CDR)^20^ were used to measure global cognition and severity of dementia, respectively. The Chinese Version Verbal Learning Test (CVVLT) (9 items; total correct trials 1–4, and 10-min delayed free recall)^21^ was administered to evaluate verbal memory performance. Total recall (i.e., the total number of items remembered over 4 trials, CVVLT-T) and free delayed recall (i.e., 10-minute delayed free recall, CVVLT-10) were analysed.

### Clinical diagnosis

Clinical diagnosis was based on physical examination, clinical interview, and neuropsychological assessment. A diagnosis of amnestic single-domain MCI was based on the criteria recommended by the National Institute on Aging/Alzheimer’s Association (NIA-AA) workgroups in 2011^22^. Episodic memory impairment was determined by a score of 1.5 standard deviations below the age- and education-matched normative mean with no accompanying impairments in social or occupational functioning, as assessed by the activities of daily living scale and the instrumental activities of daily living scale. In addition, all MCI subjects had a CDR score of 0.5.

A formal cognitive test was conducted at annual follow-up visits to ascertain the development of dementia. A diagnosis of probable AD was made when patients fulfilled the core clinical criteria proposed by the NIA-AA workgroup and had a CDR score of 1^23^. Neurological examinations, laboratory tests, and MRI were performed at baseline to exclude non-AD causes of dementia. Exclusion criteria included evidence of other neurological, psychiatric or systemic conditions that may cause cognitive impairment, such as frontotemporal dementia, dementia with Lewy bodies, stroke, vascular dementia, Parkinson’s disease, thyroid dysfunction, renal insufficiency, vitamin B12 deficiency, neurosyphilis, alcoholism, and major depression.

The MMSE score was used to measure disease progression. Subjects were dichotomized into a stable group and a declined group according to change in MMSE scores from baseline to follow-up evaluation. Patients with a decrease in MMSE score from baseline were placed in the declined group, while those with no change or an increase in MMSE score were placed in the stable group.

### Blood sample processing

Venipuncture was used to collect whole blood into ethylenediaminetetraacetic acid (EDTA)-treated tubes after overnight fasting. Blood was centrifuged (1500–2500 × *g*, room temperature, 15 min) and the supernatant was collected and divided into various aliquots which were maintained at −80 °C until the day of testing to avoid multiple freeze/thaw cycles. Frozen plasma samples were delivered on dry ice to MagQu Co., Ltd. (New Taipei City, Taiwan) for IMR assay processing. Assays were performed without knowledge of individual identification or diagnosis.

#### Genotyping

Apolipoprotein E (ApoE) genotype was determined for all participants by polymerase chain reaction amplification and restriction enzyme digestion following methods described previously^24^.

### Immunomagnetic reduction assays

The technical information and the validation accuracy of the IMR assay have been previously well described^17,18,25–27^. The selection of antibodies conjugated to the IMR reagents (MagQu Co. Ltd.; catalogue numbers: MF-AB2-0060 and MF-TAU-0060) was based on epitopes, affinity to antigens, ability to conjugate onto MagQu magnetic nanobeads, and ability to provide linearity of standard curves quantified by magnetic signal reduction. For the Aβ42 assay, 60-μl reagent (MF-AB2-0060, MagQu) was mixed with 60-μl plasma at room temperature. For the t-tau assay, 80-μl reagent (MF-TAU-0060, MagQu) was mixed with 40-μl plasma. A SQUID-based alternative current magnetosusceptometer (model XacPro-S, MagQu Co., New Taipei City, Taiwan) was used for analysis. The magnetosusceptometer detects magnetic signal changes during the course of antigen and antibody interactions, expressed as percentage reduction of immunomagnetic signals (IMR %), which are then converted to sample concentrations using values from the standard curves of the respective analytes. The reduction of oscillation detected by SQUID corresponds to the amount of analytes bound to the antibodies. Duplicates were conducted for each biomarker assay.

### Statistical analysis

All statistical analyses were performed in SPSS version 22.0 for Windows (SPSS Inc., Chicago, IL). A *p*-value < 0.05 was considered significant. All variables were analysed through non-parametric methods. For continuous variables, differences between the stable group and the declined group were detected with a Mann-Whitney U test. For categorical variables, the Chi-square test was used. Spearman’s rank correlation coefficient was used to explore the correlation between plasma biomarker levels and cognitive performance (i.e., MMSE and CVVLT). Receiver operating characteristic (ROC) analyses were computed to identify possible useful cut-off points of single Aβ42 or t-tau analytes, or their combinations (ratio or product) to further characterize discriminatory properties between the stable and declined groups. Additional analysis with Cox proportional regression (enter method) was carried out to investigate the power of biomarker levels and respective cut-off values in the prediction of cognitive decline in MCI, in terms of hazard ratios, with and without adjusting by age, gender, years of education, and ApoE ɛ4 carrier status.

## Results

### Baseline demographic data

At baseline, 22 subjects with amnestic MCI were enrolled in the study. The average follow-up period was 18.6 months (range, 9.7–25.8 months; median, 21.3 months). The demographic characteristics of the subjects are presented in Table 1. The annual conversion rate from MCI to AD was 14.7%. There were no significant differences in age, gender, years of education, proportions of ApoE ε4 carrier, or baseline MMSE scores between the stable and declined groups. The declined group had poorer baseline performance on the CVVLT, higher levels of plasma biomarkers, and greater incidence of conversion to AD during follow-up. In addition, the MMSE decreased by an average of 2.2 points per year in the declined group, while the stable group showed minimal change (0.84 point/year).

**Table 1:** Participant demographic data (N = 22).

	Stable group (N = 13)	Declined group (N = 9)	*p*
Age, years	73.0 (62.5–80.0)	76.0 (69.0–79.5)	0.547
Male	9 (69.2%)	4 (44.4%)	0.384
Education	12.0 (12.0–15.0)	12.0 (7.5–16.0)	0.780
ApoE ε4 carrier	3 (23.1%)	3 (33.3%)	0.655
MMSE	27.0 (25.0–28.0)	26.0 (25.0–26.5)	0.234
CVVLT-T	27.0 (25.8–29.0)	21.0 (15.5–22.5)	0.002**
CVVLT-10	7.0 (5.5–8.0)	1.0 (0.0–2.0)	0.002**
Conversion to AD	0 (0%)	4 (44.4%)	0.017*
Reversion to normal	1 (7.6%)	0 (0%)	1.000
Aβ42, pg/ml	16.5 (15.5–17.1)	18.8 (17.3–20.1)	0.021*
t-tau, pg/ml	18.7 (15.6–23.2)	27.2 (20.2–39.6)	0.021*
Aβ42 × t-tau, pg^2^/ml^2^	325.0 (248.1–453.7)	571.3 (373.2–778.3)	0.012*
Aβ42/t-tau	0.9 (0.7–1.1)	0.6 (0.5–1.0)	0.077

Abbreviations: n, number of participants; ApoE, apolipoprotein E; MMSE, Mini-Mental State Examination; CVVLT, Chinese Version Verbal Learning Test; t-tau, total tau.

Note: The continuous variables, presented as median values and interquartile ranges in parentheses, were calculated by a Mann-Whitney U test. The categorical variables, presented as number of patients and percentage in parentheses, were examined by a Chi-square test.

*p < 0.05, **p < 0.01.

### Association of plasma biomarkers with MMSE and CVVLT at baseline

**Figure 1:**
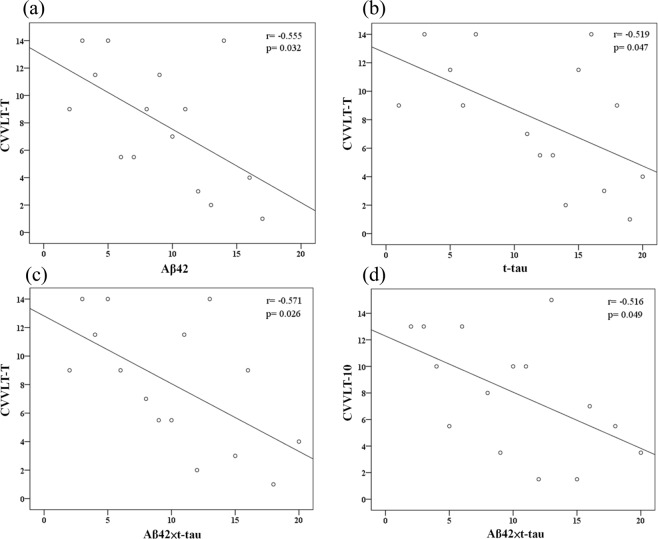
Scatterplots of the associations between baseline CVVLT scores and plasma biomarker levels (pg/ml).

There was a significant correlation between the cognitive performance on verbal memory test and plasma biomarker levels (Table 2). The CVVLT-T scores were significantly correlated with Aβ42 levels (r = −0.555, p = 0.032), t-tau (r = −0.519, p = 0.047), and the product of Aβ42 and t-tau levels (Aβ42 × t-tau, r = −0.571, p = 0.026), while the CVVLT-10 scores were significantly correlated with Aβ42 × t-tau (r = −0.516, p = 0.049) (Table 2 and Fig. 1).

**Table 2:** Association between baseline plasma biomarkers and MMSE and CVVLT scores at baseline and follow-up (N = 22).

Baseline	MMSE		CVVLT-T		CVVLT-10	
Biomarkers	r_s_	*p*	r_s_	*p*	r_s_	*p*
Aβ42	−0.024	0.917	−0.555	0.032*	−0.393	0.147
t-tau	−0.138	0.539	−0.519	0.047*	−0.512	0.051
Aβ42 × t-tau	−0.061	0.786	−0.571	0.026*	−0.516	0.049*
Aβ42/t-tau	0.160	0.478	0.499	0.058	0.491	0.063
**Annual changes**	**MMSE**		**CVVLT-T**		**CVVLT-10**	
**Biomarkers**	**r**_**s**_	***p***	**r**_**s**_	***p***	**r**_**s**_	***p***
Aβ42	−0.512	0.015*	0.009	0.975	−0.022	0.939
t-tau	−0.376	0.085	−0.070	0.805	0.005	0.985
Aβ42 × t-tau	−0.429	0.046*	−0.077	0.785	−0.029	0.919
Aβ42/t-tau	0.244	0.273	0.100	0.723	0.040	0.888

Abbreviations: MMSE, Mini-Mental State Examination; CVVLT, Chinese Version Verbal Learning Test; t-tau, total tau.

Note: Spearman’s rank correlation coefficient was used to explore the correlation between plasma biomarker levels and MMSE and CVVLT scores at baseline and follow-up.

*p < 0.05.

### Association of plasma biomarkers with annual changes in MMSE and CVVLT

**Figure 2:**
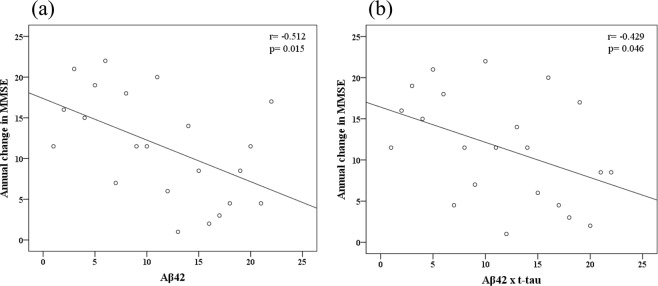
Scatterplots of the associations between annual change in MMSE scores and plasma biomarker levels (pg/ml).

There was a significant negative correlation between the annual change in MMSE scores and plasma biomarker levels (Table 2). The annual change in MMSE scores was correlated with the Aβ42 levels (r = −0.512, p = 0.015) and Aβ42 × t-tau (r = −0.429, p = 0.046) (Table 2 and Fig. 2). However, there was no significant correlation between plasma biomarkers and annual changes in CVVLT-T and CVVLT-10 scores.

### ROC analysis for prediction of cognitive decline in MCI

**Figure 3:**
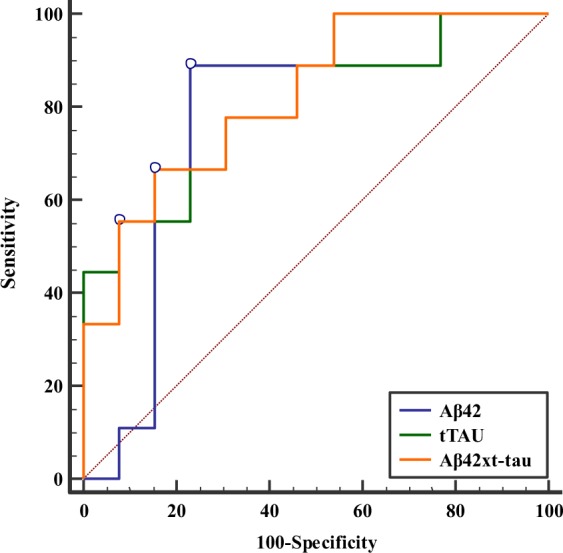
ROC curve for discriminating the stable group from the decline group using Aβ42 level, t-tau level, and Aβ42 × t-tau value as diagnostic parameters.

The discriminatory properties of plasma biomarkers for prediction of cognitive decline were investigated using ROC analyses of the stable group versus the declined group in order to obtain cut-off values at the greatest area under the ROC curves (AUC). Optimal cut-off values of 16.8 pg/ml for Aβ42, 25.4 pg/ml for t-tau, and 465.1 pg^2^/ml^2^ for Aβ42 × t-tau were identified in the differentiation of stable and declined participants. Aβ42 × t-tau obtained the highest AUC (0.82, p < 0.001). The sensitivity (SN), specificity (SP), and AUC of single Aβ42 and t-tau were enhanced by the computed product of Aβ42 and t-tau. Detailed results for AUC, cut-off values, SN, SP, accuracy (AC), and positive likelihood ratio (LR+) are displayed in Table 3 and Fig. 3.

**Table 3:** ROC analysis for cognitive decline in MCI.

Variables	AUC (95% CI)	*p*	Cutoff	SN (%)	SP (%)	AC (%)	LR+
Aβ42	0.80 (0.57–0.94)	0.005**	>16.8	88.89	76.92	81.82	3.85
t-tau	0.80 (0.57–0.94)	0.004**	>25.4	55.56	92.31	77.27	7.22
Aβ42 × t-tau	0.82 (0.60–0.95)	<0.001**	>465.1	66.67	84.62	77.27	4.33
Aβ42/t-tau	0.72 (0.49–0.88)	0.087	≤0.63	55.56	92.31	77.27	7.22

Abbreviations: AUC, area under the receiver operating characteristic curve; CI, confidence interval; SN, sensitivity; SP, specificity; AC, accuracy; LR+, positive likelihood ratio.

**p < 0.01.

### Association of plasma biomarkers with cognitive decline in MCI

The relationship between plasma biomarkers and incidence of cognitive decline during the follow-up visits was examined through Cox regression analyses using the optimal cut-offs as described above. The results of the unadjusted and adjusted Cox regression models for estimating the relative risk of developing cognitive decline in MCI patients are listed in Table 4. All markers, except Aβ42/t-tau, were significantly associated with cognitive deterioration in MCI patients. These findings were subsequently confirmed in the adjusted model. All adjusted hazard ratios (aHRs) increased after controlling for age, gender, years of education, and ApoE ɛ4 carrier status. Participants with Aβ42 > 16.8 pg/ml (aHR, 16.84; 95% CI, 1.88–150.45; p = 0.011), Aβ42 × t-tau values > 465.1 pg^2^/ml^2^ (aHR, 7.14; 95% CI, 1.57–32.55; p = 0.011), and t-tau levels >25.4 pg/ml (aHR, 5.19; 95% CI, 1.20–22.53; p = 0.028) were at increased risk for future cognitive decline.

**Table 4:** Cox regression analysis of predictors for cognitive decline in MCI.

	Unadjusted			Adjusted*		
	HR	95% CI	*p*	HR	95% CI	*p*
**Aβ42, pg/ml**						
≤16.8	1.00			1.00		
>16.8	11.71	(1.45–94.64)	0.021*	16.84	(1.88–150.45)	0.011*
**t-tau, pg/ml**						
≤25.4	1.00			1.00		
>25.4	4.51	(1.19–17.13)	0.027*	5.19	(1.20–22.53)	0.028*
**Aβ42 × t-tau, pg** ^**2**^ **/ml** ^**2**^						
≤465.1	1.00			1.00		
>465.1	6.08	(1.45–25.41)	0.013*	7.14	(1.57–32.55)	0.011*
**Aβ42/t-tau**						
≤0.63	1.00			1.00		
>0.63	0.28	(0.08–1.07)	0.063	0.29	(0.07–1.17)	0.083

Abbreviations: HR, hazard ratio; CI, confidence interval.

*Models were adjusted for age, gender, years of education, and APOE ɛ4 carrier status.

*p < 0.05.

## Discussion

The current preliminary study assessed the predictive power of plasma Aβ42 and t-tau in cognitive decline in patients with amnestic MCI. IMR technology, a high-sensitivity assay platform, was used to reliably detect ultra-low concentrations of Aβ42 and t-tau in blood. Higher levels of plasma biomarkers (i.e., Aβ42, t-tau and Aβ42 × t-tau) were found in participants who showed cognitive decline (the declined group) compared to those who did not (the stable group) and were associated with lower episodic verbal memory performance at baseline and a greater annual decrease in MMSE score. Aβ42 and t-tau had moderate-to-high discriminatory ability (AUC > 0.70), while combining the two biomarkers (i.e., Aβ42 × t-tau) added further differential value. After adjusting for relevant demographic covariates (age, gender, years of education, and ApoE ɛ4 carrier status), Aβ42, t-tau, and Aβ42 × t-tau proved to be strong predictive biomarkers for future cognitive decline in MCI. Collectively, these findings suggest that both Aβ42 and t-tau are potential predictors for monitoring progressive cognitive decline in the MCI stage of AD.

During AD progression, Aβ and tau proteins are readily excreted from the brain into the peripheral blood through disruption of blood-brain barrier and receptor-mediated mechanisms^10,12,28^. Aβ and tau are therefore detectable in plasma through highly sensitive and accurate analytic techniques. Until now, there has been great progress in the development of novel techniques to measure Aβ and tau in the blood (reviewed by others^29–32^). Recent studies using ultrasensitive analytical assays, such as IMR, single-molecule array (SIMOA), and immunoprecipitation mass spectrometry (IP-MS) approaches, have obtained enhanced technical accuracy of biomarkers in blood samples. IMR assays have the capacity to quantify plasma Aβ and tau at levels as low as 1–10 pg/ml^25,33,34^. Plasma Aβ42 levels lower than the detection limit of conventional Aβ42 enzyme-linked immunosorbent assays (ELISAs) are likely to be encountered in the MCI stage and bias parameter estimates at or below the detection threshold of conventional ELISAs (e.g., false negatives). These assays detect an increase in Aβ42 signal in prodromal AD with high accuracy (nearly 85%), and using an Aβ42 × t-tau of 382.68 pg^2^/ml^2^ as the cut-off value was 92% accurate in the identification of AD^16,18^. SIMOA assays are capable of detecting Aβ42 at subpicogram/ml levels (near 0.04 pg/ml), and have shown that Aβ42 levels and Aβ42/Aβ40 ratios are correlated with CSF levels and Aβ burden on PET and that Aβ42/Aβ40 ratios are associated with risk of MCI or AD in cognitively normal subjects^13,35^. Recent IP-MS study has demonstrated that alterations in plasma and CSF Aβ42 levels show similar kinetics and that plasma Aβ42 levels correlate with both CSF Aβ42 levels and Aβ burden as revealed by Aβ PET^12^. Conversely, another IP-MS study reported that a decrease in Aβ42 levels and Aβ42/Aβ40 ratios predicted Aβ PET positivity in AD, MCI, and cognitively normal populations with a high accuracy (nearly 90%)^14^. Moreover, a newly-developed antibody-based infrared sensor method has shown that the secondary structure change of plasma Aβ peptides correlates with CSF AD biomarkers and Aβ PET imaging^36^. The contradictory findings across the above studies may be partially explained by the various detection capacities of Aβ aggregates or Aβ bound to other proteins using different bioanalytical platforms.

Prior studies have shown t-tau levels to be significantly higher in AD than healthy controls, but there is considerable overlap across the diagnostic groups using both IMR^18^ and SIMOA^37^ methods. A previous IMR study found a negative association between plasma t-tau and volume of total gray matter, hippocampal volume, amygdale volume, and cognitive measures of logical memory, visual reproduction, and verbal fluency in MCI or early AD^26^. Recent studies using SIMOA have emphasized the role of plasma t-tau/Aβ42 in the prediction of brain tau pathology and neurodegeneration in AD and have also demonstrated a positive correlation of phospho-tau181 levels with clinical AD severity and deposition of tau and Aβ detected by PET^15,38^.

Unlike the findings from studies using conventional assay technologies, studies using ultrahigh-sensitivity assay technologies have consistently shown significant correlations between peripheral and central markers of amyloidosis and neurodegeneration indicative of brain AD pathology. Due to the differing findings reported above, further studies using larger longitudinal cohorts of participants are necessary to determine the practicability of plasma biomarkers in the clinical settings.

Studies that have explored plasma Aβ42 in the pre-dementia stage of AD have yielded inconsistent results and a broad spectrum of changes. Prior studies have shown that plasma Aβ42 levels are higher in non-demented participants with a greater risk for dementia and AD^39,40^ and in subjects with MCI, compared to healthy controls and AD patients^41–43^. Other studies have shown that plasma Aβ42 concentrations increased prior to dementia onset in familial AD with presenilin or amyloid precursor protein (APP) mutations^44,45^, in Down syndrome with *APP* triplication^46,47^, and in first-degree relatives of AD patients, who are at an increased risk of developing the disease^48–50^. Conflicting studies have found decreased plasma Aβ42 or Aβ42/Aβ40 ratio associated with the progression from healthy controls and MCI to AD and cognitive decline in a population at risk for AD^51–55^. Moreover, one study found no difference in plasma Aβ42 levels between healthy controls, subjects with stable MCI, MCI progressors, and AD patients^56^.

Studies on t-tau plasma levels have also yielded inconsistent results. For example, prior studies have shown that compared with cognitively normal controls, t-tau levels increase in AD, but not in MCI^37,57^, rise in MCI^58^, elevate in both MCI and early AD^26^, do not change in both MCI and AD^59^, and decrease in MCI and AD^60^. The discrepancies between findings for these biomarkers may be caused by varied quantification methods (e.g., digital array technology, SIMOA, IMR assay, and ELISA), platform-related factors (e.g., matrix effect, interference, and epitope masking), confounding clinical and demographic factors (e.g., age, renal/hepatic function, comorbidities, dietary status, study cohort, disease stage, and follow-up duration), and the absence of verification for brain Aβ or tau accumulation. Despite the heterogeneity of results, plasma markers of AD pathology remain a focus of interest in predicting development of cognitive decline in at-risk subjects because they are simple, inexpensive, and non-invasive, all of which are significant merits for population-based screening tools.

The present study identified significant negative associations between levels of plasma Aβ42, t-tau, and Aβ42 × t-tau and cognitive measures of episodic verbal memory and annual change in MMSE scores. These findings are in line with previous studies showing an inverse relationship between levels of Aβ42 and t-tau and cognitive performance in the pre-dementia stage^26,43,57,61–64^. In addition, at follow-up (with a mean period of 1.5 years), we found that Aβ42 levels increase in blood during the clinical phase of amnesic MCI. This finding is extremely valuable from a clinical perspective, as the ability to identify at-risk individuals 1–2 years prior to a clinical diagnosis of AD can significantly improve the quality of care and allow patients and their families to plan and to receive timely practical information and support, and to initiate suitable clinical intervention.

Both plasma Aβ42 and t-tau can serve as a window, providing insights into brain functioning involved in verbal memory and global cognition during the natural progression of amnestic MCI. The findings from the present study along with results from prior studies reviewed above, showing associations of plasma biomarkers with CSF biomarkers, neuroimaging abnormalities and cognitive measures, indicate that increased plasma Aβ42 and t-tau are likely to occur during the progressive course of MCI in parallel with cognitive decline before the clinical onset of AD. These biomarkers, therefore, reflect the pathophysiological process of AD that commences years before overt cognitive functions worsen.

ROC curves were used to determine the cut-off values for each plasma biomarker and their combination to differentiate participants in the decline group from the stable group. This analysis indicated that Aβ42 alone exhibited high SN, SP, and AC in the identification of MCI patients with cognitive decline. Higher plasma Aβ42 (>16.8 pg/ml) and t-tau (>25.4 pg/ml) at the onset of the study were associated with a 5–17-fold increased risk of cognitive decline. Since the concentrations of both Aβ42 and t-tau protein are higher in the decline group than in the stable group, it is reasonable to use the composite marker of Aβ42 × t-tau as a potential prognostic parameter to improve the discriminatory power. Single Aβ42 and t-tau had moderate-to-high predictive ability (AUC > 0.70), while the combination of the two biomarkers (i.e., Aβ42 × t-tau) showed an incremental benefit to between-group differentiation. Aβ42 × t-tau achieved a higher AUC (0.82) and acceptable SN (66.67%) and SP (84.62%). Although this combination did not increase LR+, it retained adequate predictive performance and revealed a sufficient discrimination power between the two groups (LR+ = 4.33). The cut-off values for levels of Aβ42 and t-tau used in the current study to differentiate groups are in agreement with other studies performed with non-demented elderly subjects^16–18^.

After controlling for the demographic covariates, Aβ42, t-tau, and Aβ42 × t-tau showed strong promise as predictive biomarkers for future cognitive decline in the MCI stage, whereas Aβ42/t-tau ratio did not have predictive value. Using SIMOA and xMAP technology, Aβ42, which positively correlated with CSF Aβ42 but negatively with CSF t-tau, was higher in the MCI stage than in the AD stage, while t-tau was positively associated with the burden of brain tau deposition on tau PET across the AD spectrum, that is, Aβ42 elevates during the MCI stage and reaches a plateau before the demented stage, while t-tau increases with AD–associated tau pathology^15,43^. Therefore, the product of Aβ42 and t-tau improved the clinical usefulness in the MCI stage, which was supported by AUC analyses of predicting accuracy for developing cognitive decline. We consider that utilization of plasma Aβ42 and t-tau together is superior to absolute levels of individual peptides and to the Aβ42/t-tau ratio as markers of clinical deterioration in MCI. As this study evaluated a short time period of about 1.5 years in a small cohort of 22 subjects with MCI, our preliminary data might only infer a possible association of plasma Aβ42 and t-tau with an imminent risk of conversion to AD. This result warrants a further replication with a larger sample over a longer time period to validate and determine their long-term predictive value in detecting AD. Further longitudinal studies are needed to assess whether high plasma Aβ42 and t-tau are clinically useful prognostic biomarkers for AD. Whether the plasma biomarkers alone, in combination, or with other biomarkers, improve diagnostic efficiency, patient discrimination, and disease monitoring along the normal to AD continuum deserves additional researches.

While the current study contributes valuable findings, there are some limitations that should be considered. First, the sample size was relatively small. A larger, more diverse population with a longer follow-up period is needed to support the generalization of these results and to establish the significance of plasma biomarkers in the identification of individuals at risk for cognitive deterioration in the MCI stage of AD. Second, a larger sample size is required to validate the measures of sensitivity, specificity, and accuracy in the ROC analysis as well as obtain the optimal cut-off values of Aβ42 and t-tau. Third, MCI duration was not controlled for in the present study and may have affected plasma biomarker levels. Forth, the current study did not perform Aβ PET or provide neuropathological confirmation of dementia. Finally, comorbidities that may affect plasma biomarker levels were not covaried in the present study.

In conclusion, consistent with previous studies showing promising discriminatory power of plasma Aβ42 and t-tau assays with IMR technology over the normal–MCI–AD cognitive spectrum^16–18^, the overall findings of the current preliminary study might support that higher plasma Aβ42 and t-tau levels in the MCI stage are predictors of greater risk for the development of cognitive decline in the pre-dementia stage of AD. Aβ42 and t-tau might therefore be considered as markers reflecting disease severity in monitoring patients with early memory disorders. Combining the two plasma biomarkers with other markers might help to identify MCI subjects at risk for AD. The early detection of patients at risk will be beneficial for early and preventive pharmacological intervention.
